# Characterization of microvascular tortuosity in retinal vein occlusion utilizing optical coherence tomography angiography

**DOI:** 10.1038/s41598-020-74871-7

**Published:** 2020-10-20

**Authors:** Hyungwoo Lee, Myung Ae Kim, Hyung Chan Kim, Hyewon Chung

**Affiliations:** Department of Ophthalmology, Konkuk University School of Medicine, Konkuk University Medical Center, 120-1 Neungdong-ro, Gwangjin-gu, Seoul, 05030 Republic of Korea

**Keywords:** Eye diseases, Prognostic markers

## Abstract

We investigated the characteristics of microvessel tortuosity in branch retinal vein occlusion (BRVO) and central retinal vein occlusion (CRVO) and their associations with visual outcomes using optical coherence tomography angiography (OCTA). Thirty-four BRVO and 21 CRVO patients and 31 healthy subjects were included. From OCTA, the branch number (BN), mean branch length (BL), mean Euclidean length (EL), vessel density (VD) and vessel tortuosity (VT) were quantified. In BRVO eyes, compared with that in the controls, the affected area of the deep capillary plexus (DCP) showed a decreased BN and VD, an increased BL, and unchanged VT. The nonaffected area of the DCP showed decreases in BN, VD and VT. The affected area of the superficial capillary plexus (SCP) showed higher VT. In CRVO eyes, the DCP showed a lower BN, VD and VT, while the SCP showed a lower BN and greater BL and EL. Improved visual acuity (VA) after 1 year in BRVO eyes was associated with decreases in BN, BL, VD and VT in the affected area in the DCP and lower VT in the nonaffected area of the SCP; in CRVO eyes, improved VA was associated with a higher BL and EL in the DCP. VT, BL, and EL may be new microvascular markers associated with changes in VA in BRVO and CRVO.

## Introduction

Retinal vein occlusion (RVO) is the second most common retinal vascular disease after diabetic retinopathy, and it is considered to be an important cause of visual impairment. The development of macular edema (ME) causes visual deterioration and is thought to be caused by venous stasis and increased vascular permeability in RVO^1^. However, the severity of ME is not always correlated with visual prognosis, and their association is still debatable^2,3^. Optical coherence tomography angiography (OCTA) can provide images of the retinal microvasculature without invasive procedures. In RVO, nonperfused areas could be clearly demonstrated, and decreased vessel density (VD) on OCTA was correlated with poor visual acuity (VA)^4^. In particular, the degree of decreased VD in the deep capillary plexus (DCP), representing ischemia at the level of the DCP, has been associated with ME development and visual prognosis^5^.

Meanwhile, vessel tortuosity is another intrinsic characteristic vascular change in RVO. It represents increased venous blood stasis and a subsequent increase in transmural pressure. Increased venous tortuosity of large vessels in RVO is known to decrease after anti-vascular endothelial growth factor (VEGF) treatment^6^, suggesting that venous tortuosity reflects the degree of severity of venous occlusion in RVO. However, microvascular changes rather than large vascular changes in RVO are more likely to reflect the degree of capillary ischemia derived from vein occlusion and are thus associated with the clinical course and long-term visual prognosis. Vessel tortuosity (VT) at the microvascular level has not been quantified in RVO, and its clinical significance is also unknown.

Thus, in the present study, we aimed to reveal new vascular biomarkers reflecting capillary ischemia resulting from venous stasis in RVO using OCTA. To achieve this goal, the detailed characteristics of microvascular changes in RVO, such as the branch number, branch length (BL), Euclidean length (EL), VT and VD, were calculated in both affected and fellow eyes in the BRVO and CRVO groups and in the control group. Finally, these parameters were correlated with VA 1 year after initial treatment.

## Results

A total of 86 eyes from 86 subjects were included in this study: BRVO patients (34 eyes of 34 patients), CRVO patients (21 eyes of 21 patients) and healthy control subjects (31 eyes of 31 patients). The characteristics of the participants are shown in Table 1.

**Table 1:** Clinical characteristics of the study groups.

	Control eyes	BRVO fellow eyes	BRVO eyes	CRVO fellow eyes	CRVO eyes	P
No. of patients (n)	31	34	34	21	21	N/A
No. of eyes (n)	31	34	34	21	21	N/A
Sex (male/female)	7/24	15/19	15/19	12/9	12/9	0.03
Age (year, mean ± SD)	56.9 ± 10.1	59.3 ± 10.8	59.3 ± 10.8	62.2 ± 15.9	62.2 ± 15.9	0.30
DM, n (%)	0 (0)	5 (14.7)	5 (14.7)	11 (52.4)	11 (52.4)	0.01
HTN, n (%)	0 (0)	16 (47.1)	16 (47.1)	12 (57.1)	12 (57.1)	0.58
Interval from the first treatment to the day of OCTA imaging (months)	N/A	1.8 ± 4.4	2.8 ± 2.2	2.9 ± 4.4	3.6 ± 2.9	0.28
No. anti-VEGF injections in the first year	N/A	N/A	2.4 ± 1.8	N/A	2.9 ± 1.9	0.37
Baseline BCVA	N/A	0.024 ± 0.076	0.371 ± 0.489	0.043 ± 0.063	1.017 ± 0.801	< 0.001
BCVA on the day of OCTA imaging	0.006 ± 0.029	0.019 ± 0.059	0.221 ± 0.311	0.070 ± 0.129	0.400 ± 0.517	< 0.001
BCVA after 1 yr from baseline	N/A	0.077 ± 0.110	0.189 ± 0.286	0.289 ± 0.284	0.693 ± 0.907	0.11
CMT	262.19 ± 22.65	261.18 ± 21.90	298.18 ± 62.47	269.48 ± 22.90	309.00 ± 77.90	< 0.001

All values are presented as the mean ± standard deviation.

A chi-square test was performed to compare the ratios of sex, DM and HTN among the five studied groups. Differences in the mean values of other parameters among the five groups including age, disease duration, the number of injections, VA and CMT were compared by analysis of variance (ANOVA). The number of injections between the BRVO eye and CRVO eye group was compared by the Mann–Whitney test. P < .05 was considered statistically significant.

*Control* the right eyes of healthy subjects; *BRVO eyes* eyes diagnosed with branch retinal vein occlusion; *CRVO eyes* eyes diagnosed with central retinal vein occlusion; *BRVO fellow* contralateral eye of the eye diagnosed with BRVO; *affected* affected half area of the OCTA image where the vein is occluded in BRVO (in *fellow eyes* affected refers to the contralateral area of the corresponding affected area of the occluded eye); *nonaffected* affected half area of the OCTA image where the vein is not occluded in BRVO (in fellow eyes, nonaffected refers to the contralateral area of the corresponding nonaffected area of the occluded eye); *DM* diabetes mellitus; *HTN* hypertension; *OCTA* optical coherence tomography angiography; *VEGF* vascular endothelial growth factor; *BCVA* best-corrected visual acuity presented as the logarithm of the minimum angle of resolution; *CMT* central macular thickness; *N/A* not applicable.

### Comparison of parameters between superior and inferior areas in the same eye

When comparing the superior and inferior halves of the retina in both the SCP and DCP from control, CRVO, and CRVO fellow eyes, no significant difference in any measured parameters was observed (Supplemental Table S1). Additionally, no significant difference was observed between the superior and inferior areas in either the SCP or DCP of the fellow eyes of BRVO patients (Table 2). When comparing the affected area and nonaffected area in the DCP of BRVO eyes, VT, mean BL, and mean EL were higher and branch number was lower in the affected area (Table 2). In the SCP of BRVO eyes, the affected area showed higher VT (Table 2).

**Table 2:** Microvascular parameter values of the studied eyes.

SCP	Control eyes (N = 31)	BRVO fellow eyes affected (N = 34)	BRVO fellow eyes nonaffected (N = 34)	BRVO eyes Affected (N = 34)	BRVO eyes nonaffected (N = 34)	CRVO fellow eyes (N = 21)	CRVO eyes (N = 21)
**SCP**							
Branch number	293.871 ± 90.195	292.235 ± 108.897	303.882 ± 114.693	260.618 ± 85.151	267.265 ± 101.579	279.238 ± 101.88	250.857 ± 94.882
Sum of branch lengths (mm)	32.852 ± 7.762	32.965 ± 9.429	33.857 ± 9.73	30.824 ± 7.832	31.003 ± 8.626	32.049 ± 9.019	30.221 ± 9.500
Sum of Euclidean lengths (mm)	28.742 ± 6.642	28.853 ± 7.948	29.518 ± 8.172	26.706 ± 6.554	27.049 ± 7.210	27.970 ± 7.595	26.289 ± 8.171
Mean branch length (µm)	114.291 ± 10.124	118.134 ± 17.388	117.414 ± 20.099	122.688 ± 17.180*	121.454 ± 17.630	119.368 ± 15.760	126.491 ± 20.766*
Mean Euclidean length (µm)	100.181 ± 9.247	103.858 ± 16.179	102.989 ± 19.216	106.714 ± 16.052	106.477 ± 16.702	104.524 ± 14.436	110.445 ± 19.303*
Vessel tortuosity	1.142 ± 0.013	1.139 ± 0.017	1.143 ± 0.019	1.152 ± 0.019*^†^	1.143 ± 0.020	1.143 ± 0.018	1.147 ± 0.016
Vessel density	0.231 ± 0.044	0.227 ± 0.053	0.229 ± 0.056	0.215 ± 0.047	0.216 ± 0.048	0.223 ± 0.050	0.213 ± 0.059
**DCP**							
Branch number	445.065 ± 91.663	450.000 ± 94.186	450.382 ± 89.828	318.588 ± 127.817*^†^	365.235 ± 126.556*^†^	441.667 ± 71.633	282.429 ± 103.912*^†^
Sum of branch lengths (mm)	35.985 ± 9.069	36.681 ± 8.928	36.824 ± 8.946	27.045 ± 11.496*^†^	29.478 ± 11.801*^†^	35.911 ± 6.803	23.457 ± 9.672*^†^
Sum of Euclidean lengths (mm)	29.809 ± 7.141	30.533 ± 7.107	30.697 ± 7.148	22.428 ± 9.379*^†^	24.723 ± 9.572*^†^	29.726 ± 5.393	19.696 ± 8.001*^†^
Mean branch length (µm)	80.006 ± 5.665	81.029 ± 4.042	81.064 ± 5.129	83.407 ± 7.536*	78.526 ± 8.152	81.069 ± 3.717	80.980 ± 10.077
Mean Euclidean length (µm)	66.480 ± 3.964	67.592 ± 3.148	67.744 ± 3.735	69.480 ± 5.774*	66.306 ± 5.828	67.223 ± 3.255	68.225 ± 8.163
Vessel tortuosity	1.203 ± 0.024	1.199 ± 0.021	1.197 ± 0.021	1.201 ± 0.028	1.183 ± 0.028*^†^	1.207 ± 0.021	1.187 ± 0.018*^†^
Vessel density	0.204 ± 0.043	0.203 ± 0.041	0.204 ± 0.039	0.151 ± 0.057*^†^	0.165 ± 0.057*^†^	0.204 ± 0.030	0.136 ± 0.051*

All values are presented as the mean ± standard deviation.

*Control* the right eyes of healthy subjects; *BRVO eyes* eyes diagnosed with branch retinal vein occlusion; *CRVO eyes* eyes diagnosed with central retinal vein occlusion; *fellow* contralateral eye of the eye diagnosed with RVO; *affected* affected half area of the OCTA image where the vein is occluded in BRVO (in fellow eyes, affected refers to the contralateral area of the corresponding affected area of the occluded eye); *nonaffected* affected half area of the OCTA image where the vein is not occluded in BRVO (in fellow eyes, nonaffected refers to the contralateral area of the corresponding nonaffected area of the occluded eye); *SCP* superficial capillary plexus;* DCP* deep capillary plexus.

*Denotes statistically significant changes in each area compared to that of healthy eyes by Student’s t-tests.

^†^Denotes statistically significant changes in occluded eyes compared to fellow eyes by paired t-tests.

### Differences in parameters between RVO eyes and fellow eyes

**Figure 1:**
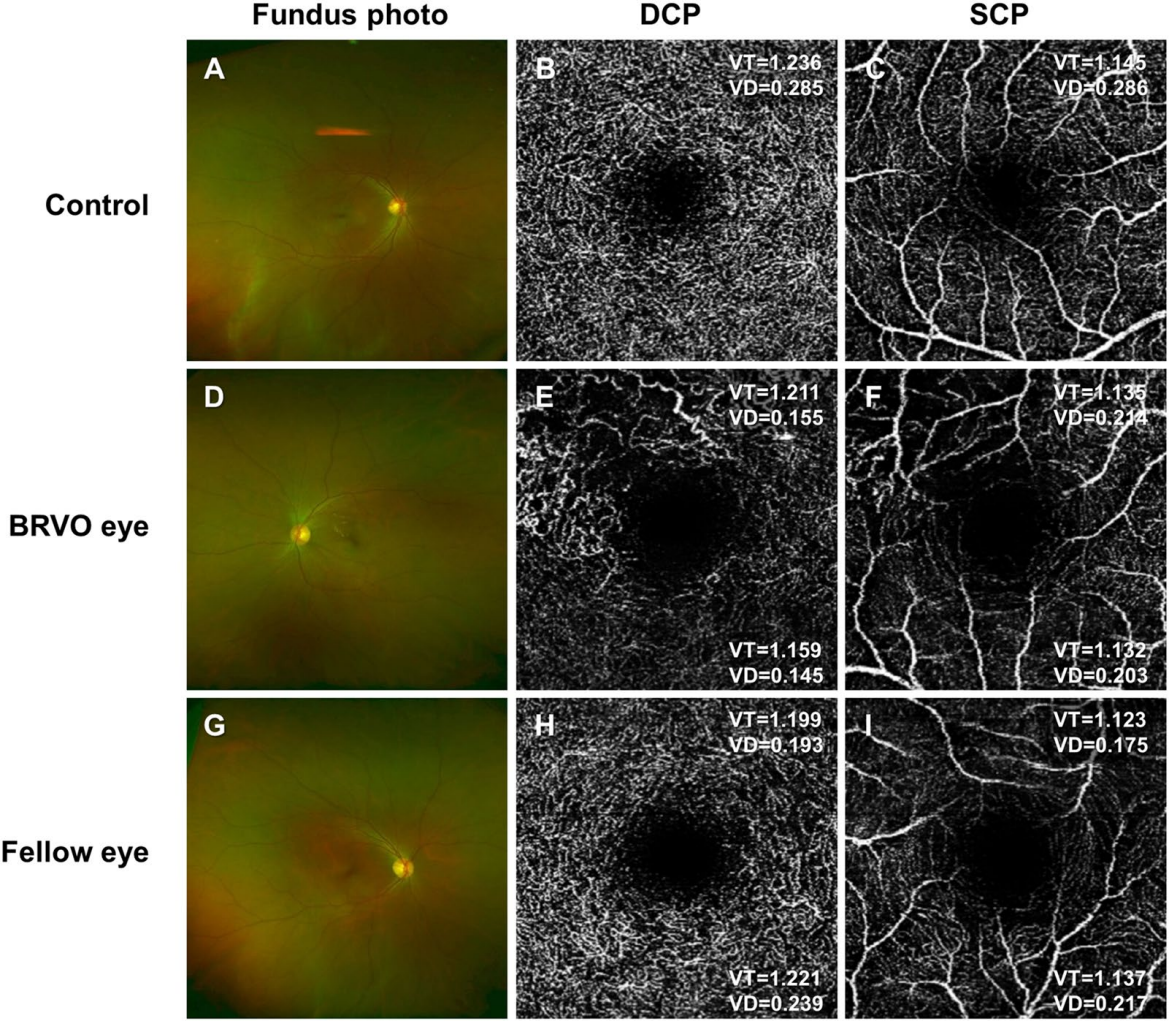
Representative images of eyes from the control and branch retinal vein occlusion (BRVO) groups. The control eye is from a 50-year-old woman whose visual acuity was 20/20. The BRVO eye is from a 56-year-old man whose visual acuity at baseline was 6/20. After 4 injections of bevacizumab, visual acuity was improved to 20/20 after 1 year. **(A–C)** Vessels in the deep capillary plexus (DCP) and superficial capillary plexus (SCP) are evenly distributed. **(D–F)** Vessel density (VD) in the DCP of the affected area (upper half of the DCP) is decreased, but vessel tortuosity (VT) is not significantly decreased. On the other hand, in the nonaffected area of the DCP (lower half of the DCP), both VT and VD are decreased. **(G–I)** In the fellow eye, VT in the affected and nonaffected areas is not significantly decreased. VD in these areas also did not decrease. *DCP* deep capillary plexus, *SCP* superficial capillary plexus, *Control* right eye of healthy subjects, *BRVO eye* eye diagnosed with branch retinal vein occlusion, *VT* vessel tortuosity, *VD* vessel density, VT and VD at the upper side in an OCTA image = VT and VD in the superior half area, VT and VD at the lower side in an OCTA image = VT and VD in the inferior half area.

**Figure 2:**
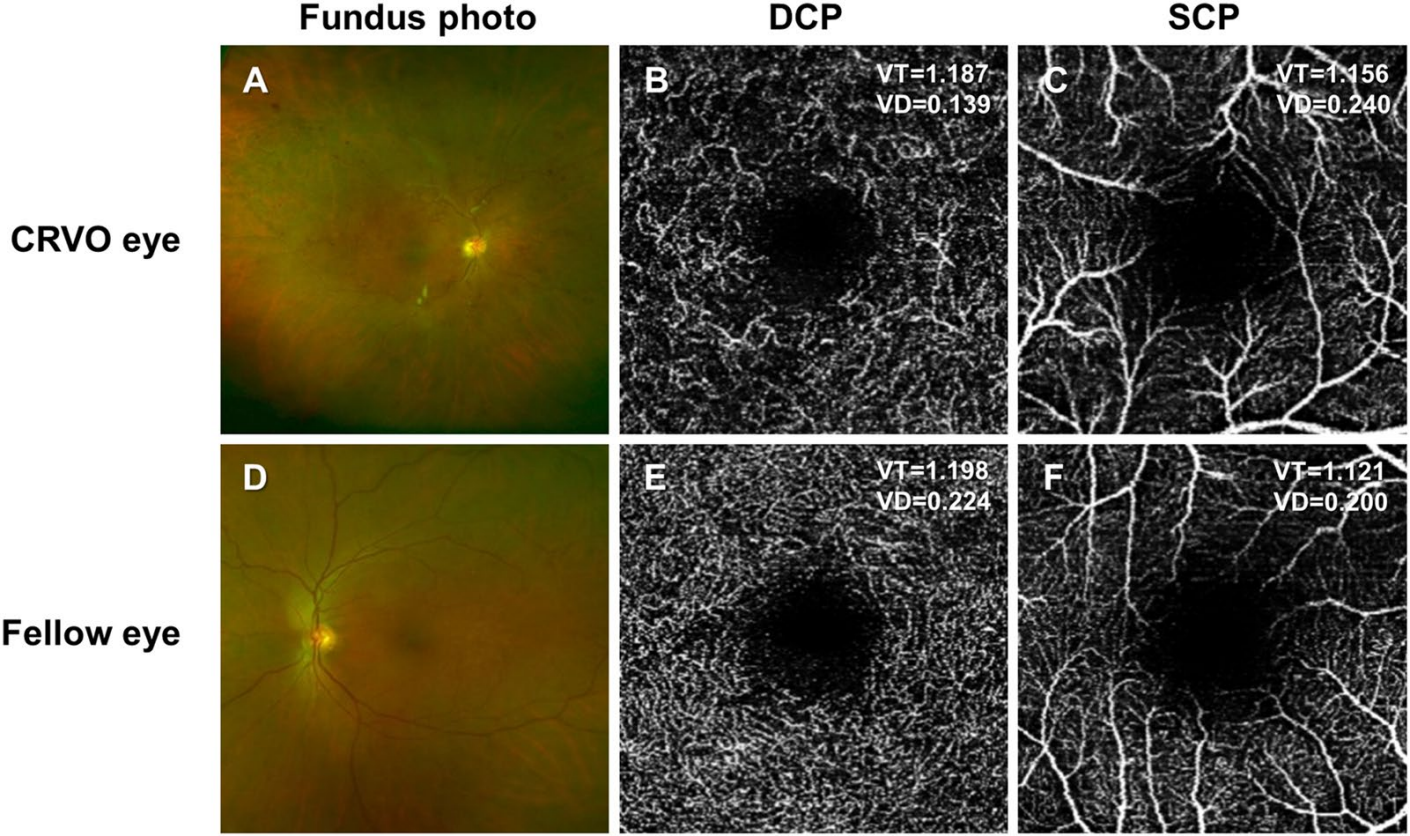
Representative images of eyes from the CRVO group. The CRVO eye is from 58-year-old woman whose visual acuity was 2/20 at baseline and improved to 8/20 after 1 year with 7 injections of ranibizumab. **(A–C)** Both VT and VD in the DCP are decreased in the investigated area (superior half area of the image), while the SCP parameters are not significantly changed. **(D–F)** In the fellow eye, VT and VD were not significantly decreased in the DCP or SCP. *DCP* deep capillary plexus, *SCP* superficial capillary plexus, *CRVO eye* eye diagnosed with central retinal vein occlusion, *VT* vessel tortuosity, *VD* vessel density, VT/VD at the upper side in an OCTA image = VT/VD in the superior half area, VT and VD at the upper side in an OCTA image = VT and VD in the superior half area, VT and VD at the lower side in an OCTA image = VT and VD in the inferior half area.

The difference between eyes with vein occlusion and fellow eyes in BRVO and CRVO patients showed a pattern similar to that of the difference between eyes with vein occlusion and the control eyes (Table 2, Fig. 1). Interestingly, the mean BL and mean EL of the affected area of the DCP in BRVO eyes were not different from those in fellow eyes, while these factors showed differences compared to the control groups (Table 2). Similarly, the mean BL and mean EL of the SCP in CRVO eyes were not different from those in fellow eyes, while these factors showed differences compared to the control groups (Table 2, Fig. 2).

### Changes in parameters in affected eyes and fellow eyes compared to the control group

In BRVO eyes, the SCP of the affected areas showed higher VT without any change in other parameters compared to the control group (Table 2, Fig. 1). The SCP of the nonaffected area in BRVO eyes showed no changes in parameters (Table 2, Fig. 1).

The DCP of the affected area in BRVO eyes showed lower branch number, sum BL, sum EL, and VD, higher mean BL and mean EL and no changes in VT compared to the control group (Table 2, Fig. 1). The DCP of the nonaffected area in BRVO eyes also showed lower branch number, sum BL, sum EL, and VD. The VT of the DCP in the nonaffected area was lower than that in the control group (Table 2, Fig. 1). In the fellow eyes of BRVO patients, no changes in parameters were observed compared to the control groups in either the SCP or DCP (Table 2, Fig. 1).

In the CRVO eyes, the SCP showed a lower branch number and higher mean BL and mean EL than control eyes, while the DCP showed lower branch number, sum BL, sum EL, VT and VD (Table 2, Fig. 2). In the fellow eyes of CRVO patients, no changes in parameters were observed compared to the control groups in either the SCP or DCP (Table 2, Fig. 2).

### Factors associated with VA

First, factors associated with VA on the day of the OCTA examination were analyzed. In the univariate regression analyses of BRVO eyes, better VA on the examination day was associated with higher branch number, sum BL, sum EL, and VD of the affected area of the DCP (Table 3). The nonaffected area of the DCP and all areas of the SCP showed no association with VA. In CRVO eyes, better VA on the examination day was associated with higher branch number, sum BL, sum EL, and VD of the DCP (Table 3). In the SCP of CRVO eyes, no factor significantly associated with VA was found.

**Table 3:** Univariate linear regression analysis of parameters associated with visual acuity on the day of the OCTA examination.

	BRVO eyes affected DCP		BRVO eyes affected SCP		BRVO eyes nonaffected DCP		BRVO eyes nonaffected SCP		CRVO eyes DCP		CRVO eyes SCP	
	Beta	P	Beta	P	Beta	P	Beta	P	Beta	P	Beta	P
Total branch numbers	− 0.13	0.003	− 0.09	0.18	− 0.08	0.12	− 0.05	0.36	− 0.38	< 0.001	− 0.30	0.06
Sum of branch length (mm)	− 0.15	0.002	− 0.12	0.10	− 0.07	0.16	− 0.06	0.34	− 0.34	0.002	− 0.29	0.07
Sum of Euclidean length (mm)	− 0.19	0.002	− 0.15	0.08	− 0.09	0.15	− 0.07	0.37	− 0.41	0.002	− 0.35	0.06
Mean branch length (µm)	− 0.13	0.084	0.002	0.96	− 0.03	0.68	0.001	0.98	− 1.30	0.26	0.09	0.17
Mean Euclidean length (µm)	− 0.18	0.07	− 0.002	0.95	− 0.04	0.67	0.004	0.90	− 0.14	0.32	0.08	0.23
Vessel tortuosity	− 0.71	0.73	2.69	0.36	− 0.58	0.79	− 3.49	0.23	− 10.70	0.10	9.21	0.25
Vessel density	− 2.96	0.002	− 1.97	0.10	− 1.32	0.21	− 0.91	0.44	− 6.77	0.001	− 4.48	0.06

Age and sex were adjusted.

The units for branch length sum and Euclidean length sum were adjusted to cm.

The unit for branch number was adjusted to 100 branches.

The mean of each branch length was adjusted to 10 µm.

*BRVO eyes* eyes diagnosed with branch retinal vein occlusion; *CRVO eyes* eyes diagnosed with central retinal vein occlusion; *affected* affected half area of the OCTA image where the vein is occluded in BRVO; *nonaffected* affected half area of the OCTA image where the vein is not occluded in BRVO; *SCP* superficial capillary plexus; *DCP* deep capillary plexus.

Next, factors associated with VA at 1 year from initial treatment were analyzed. In the univariate regression analyses of BRVO eyes, better VA was associated with higher branch number, sum BL, sum EL, mean BL, mean EL, and VD of the affected area of the DCP (Table 4). The nonaffected area of the DCP and all areas of the SCP showed no association with VA. In CRVO eyes, better VA was associated with a higher branch number, sum BL, sum EL, and VD in both the DCP and SCP (Table 4). Additionally, better VA was associated with higher mean BL and mean EL in the DCP and lower mean BL and mean EL in the SCP of CRVO eyes.

**Table 4:** Univariate linear regression analysis of parameters associated with visual acuity at 1 year after initial treatment.

	BRVO eyes affected DCP		BRVO eyes affected SCP		BRVO eyes nonaffected DCP		BRVO eyes Nonaffected SCP		CRVO eyes DCP		CRVO eyes SCP	
	Beta	P	Beta	P	Beta	P	Beta	P	Beta	P	Beta	P
Total branch numbers	− 0.19	0.003	− 0.15	0.09	− 0.09	0.17	− 0.07	0.31	− 0.61	0.003	− 0.99	0.02
Sum of branch length	− 0.22	0.002	− 0.17	0.06	− 0.08	0.28	− 0.09	0.27	− 0.68	0.004	− 0.93	0.02
Sum of Euclidean length	− 0.27	0.001	− 0.21	0.05	− 0.11	0.25	− 0.11	0.27	− 0.83	0.004	− 1.07	0.02
Mean branch length	− 0.47	0.004	0.06	0.51	0.02	0.89	0.03	0.58	− 0.75	0.01	0.35	0.02
Mean Euclidean length	− 0.67	0.001	0.06	0.52	0.02	0.93	0.03	0.54	− 0.93	0.01	0.37	0.03
Vessel tortuosity	− 0.20	0.95	− 0.54	0.90	0.71	0.86	− 3.68	0.40	− 33.70	0.11	6.75	0.87
Vessel density	− 4.22	0.002	− 2.70	0.09	− 1.55	0.32	− 1.47	0.36	− 13.00	0.003	− 13.80	0.02

Age and sex were adjusted.

The units for branch length sum and Euclidean length sum were adjusted to cm.

The unit for branch number was adjusted to 100 branches.

The mean of each branch length was adjusted to 10 µm.

*BRVO eyes* eyes diagnosed with branch retinal vein occlusion; *CRVO eyes* eyes diagnosed with central retinal vein occlusion; *affected* affected half area of the OCTA image where the vein is occluded in BRVO; *nonaffected* affected half area of the OCTA image where the vein is not occluded in BRVO; *SCP* superficial capillary plexus; *DCP* deep capillary plexus.

Finally, factors associated with the VA change 1 year after initial treatment were analyzed. In BRVO eyes, improvement of VA from the initial treatment was associated with lower branch number, sum BL, sum EL, mean BL, VT, and VD of the affected area in the DCP and lower VT of the nonaffected area in the SCP. In CRVO eyes, VA improvement was associated with a higher mean BL and mean EL of the DCP (Table 5).

**Table 5:** Univariate linear regression analysis of parameters associated with a visual acuity change at 1 year after initial treatment.

	BRVO eyes affected DCP		BRVO eyes affected SCP		BRVO eyes nonaffected DCP		BRVO eyes nonaffected SCP		CRVO eyes DCP		CRVO eyes SCP	
	Beta	P	Beta	P	Beta	P	Beta	P	Beta	P	Beta	P
Total branch numbers	0.30	0.002	0.27	0.06	0.13	0.22	0.17	0.12	− 0.25	0.27	− 0.53	0.19
Sum of branch length	0.35	0.001	0.26	0.08	0.13	0.27	0.21	0.11	− 0.33	0.20	− 0.46	0.25
Sum of Euclidean length	0.41	0.002	0.31	0.07	0.17	0.26	0.24	0.13	− 0.40	0.20	− 0.50	0.27
Mean branch length	0.62	0.03	− 0.23	0.07	0.16	0.50	− 0.65	0.43	− 0.59	0.02	0.22	0.12
Mean Euclidean length	0.51	0.18	− 0.24	0.09	0.22	0.53	− 0.08	0.35	− 0.76	0.01	0.25	0.11
Vessel tortuosity	8.50	0.04	1.37	0.84	4.03	0.51	13.30	0.04	− 13.70	0.47	− 38.50	0.23
Vessel density	6.70	0.002	4.25	0.09	2.31	0.35	3.76	0.13	− 5.79	0.24	− 5.70	0.35

Age and sex were adjusted.

The units for branch length sum and Euclidean length sum were adjusted to cm.

The unit for branch number was adjusted to 100 branches.

The mean of each branch length was adjusted to 10 µm.

*BRVO eyes* eyes diagnosed with branch retinal vein occlusion; *CRVO eyes* eyes diagnosed with central retinal vein occlusion; *affected* affected half area of the OCTA image where the vein is occluded in BRVO; *nonaffected* affected half area of the OCTA image where the vein is not occluded in BRVO; *SCP* superficial capillary plexus; *DCP* deep capillary plexus.

### Correlations between factors

In the DCP, the mean BL was positively correlated with branch number, VD and VT in the control group and in both affected and nonaffected areas of BRVO eyes (Supplementary Table S2). In the DCP of CRVO eyes, the mean BL was not correlated with VT (Supplementary Table S2).

In the SCP, the mean BL was negatively correlated with branch number, VD and VT in both affected and nonaffected areas of BRVO eyes and CRVO eyes (Supplementary Table S2).

## Discussion

In this study, we investigated the microvascular changes in RVO and their association with VA.

In the DCP, the VD in both the affected and nonaffected areas of BRVO eyes and CRVO eyes was lower than that of control eyes. In the SCP, the VD in these 3 groups also showed a decreasing tendency compared to the control group, but statistical significance was not found. Concordantly, a previous study reported that nonperfusion areas were more frequent in the DCP than in the SCP^8^. Therefore, nonperfusion might mainly occur in the DCP in RVO. Interestingly, a nonperfused area was also observed in the nonaffected area of the BRVO eyes, indicating that vein occlusion in the superior or inferior area also influences perfusion on the other side of the retina. However, VD alone cannot explain the detailed morphologic changes of vessel branches because it represents only the total area of perfusion. Thus, we further investigated VT and related comprehensive morphologic changes in vessel branches.

Tortuous venules are prominent findings in fundus photography or fluorescein angiography in RVO and are regarded as representing the degree of venous stasis^6^. In our study, unexpectedly, the VT of the nonaffected area of the DCP in BRVO eyes and CRVO eyes was lower than that in the control group. Reduced blood flow at the macula, represented as a reduced branch number in our results, might reduce transmural pressure in the branches and result in a subsequent decrease in VT. Therefore, although the decreased VT in the DCP, as observed on OCTA, and the increased tortuosity in large vessels, as observed on fundus photography, appear to be contradictory to each other, they imply that lower perfusion at the level of the DCP in the macula and venous stasis in the larger vessels can occur simultaneously or in succession.

**Figure 3:**
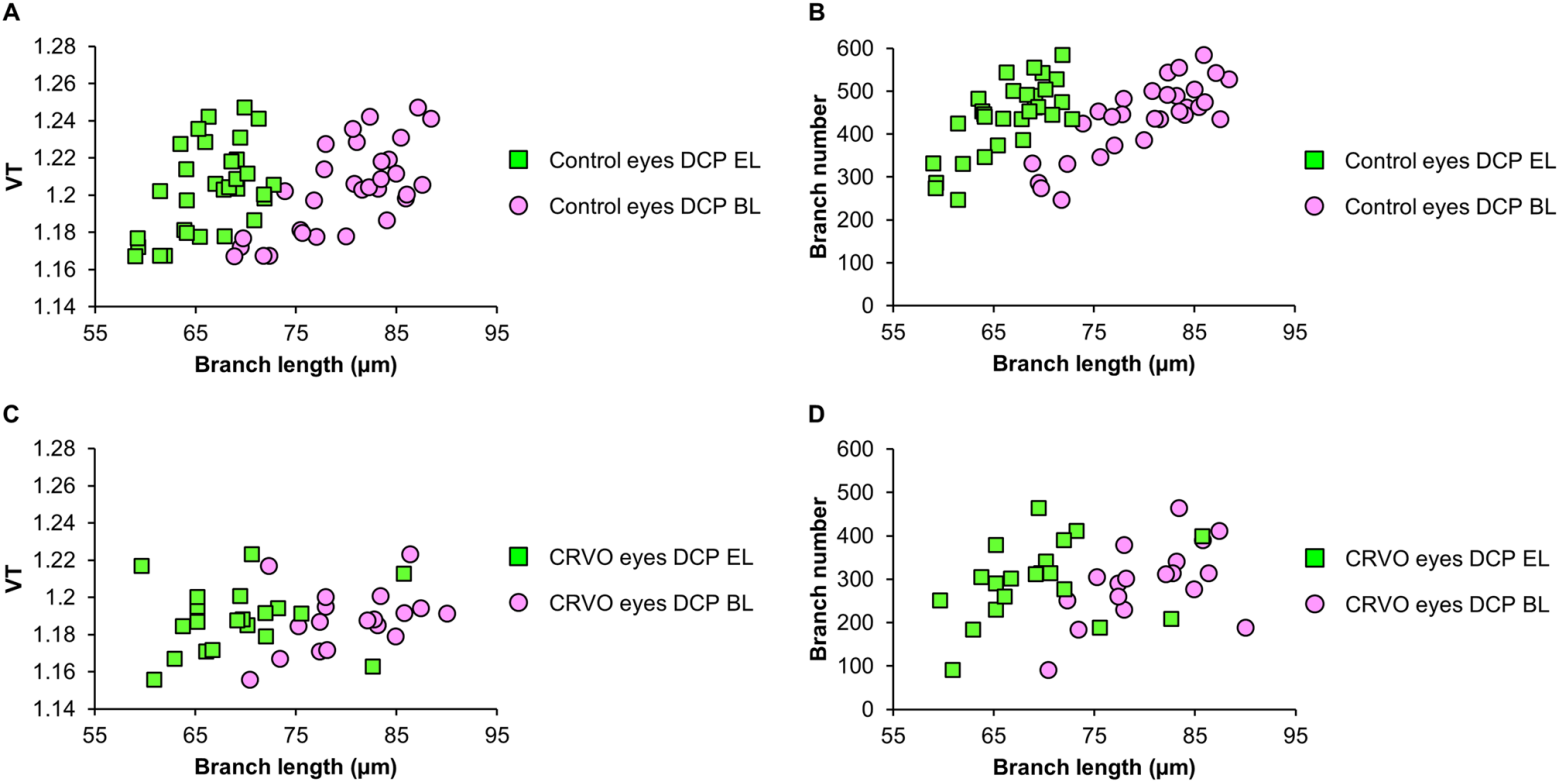
Correlations between the morphologic parameters of vessels. **(A)** Scatter plot between VT and the length of branches in the DCP of control eyes. **(B)** Scatter plot between branch number and branch length in the DCP of control eyes. **(C)** Scatter plot between VT and branch length in the DCP of CRVO eyes. **(D)** Scatterplot between branch number and branch length in the DCP of CRVO eyes. Pink circles = branch lengths, Green circles = Euclidean lengths, *VT* vessel tortuosity, *DCP* deep capillary plexus.

From the hypothesis that decreased VT is derived from reduced blood flow at the macula, VT in RVO eyes was anticipated to be lower than that in the control group. However, VT in the DCP was lower in the nonaffected area of BRVO and CRVO eyes than that in control eyes, whereas in the affected area of BRVO eyes, no change in VT compared to control eyes was found. To explain these results, which are intuitively hard to understand, we decomposed the vessels into small branches and calculated the total branch number, mean BL, and mean EL because VT and VD are representative of the overall outcomes derived from the changes in small branches. Notably, in the affected area of BRVO eyes, the mean BL and mean EL were higher than those of the control group, while branch number was reduced, indicating the prominent dropout of shorter branches. A longer BL showed a tendency toward higher VT based on their good correlation in the DCP of control eyes (r = 0.70, P < 0.001) (Fig. 3, Supplementary Table S2). The mechanism of this positive relationship between longer BL and higher VT is not clear. We speculate that increased transmural pressure derived from increased blood flow in branches with a longer BL might lead to an increase in VT. Thus, preferential loss of shorter branches left capillaries with a longer BL and thus might mask the decreased VT from hypoperfusion in the affected area of BRVO eyes. On the other hand, the nonaffected area of BRVO eyes and CRVO eyes showed no difference in mean BL or EL but reduced VD, indicating dropout of capillaries regardless of the BL or EL.

In the SCP, VT was higher in the affected area of BRVO eyes than in the control group. Based on the significant reduction of BL and VD in the DCP, microvascular occlusion might occur mainly in the DCP, and increased resistance in the DCP eventually increases transmural pressure in the SCP^9^, leading to an increase in VT in the SCP. Interestingly, an increase in VT in the SCP was not observed in the nonaffected areas of BRVO eyes and CRVO eyes. A status prone to an increase in VT—a relatively increased mean BL and no change in mean EL—was observed only in the affected area of BRVO eyes. Mean BL and EL were not changed in the nonaffected area of BRVO eyes, and these two factors increased simultaneously in CRVO eyes. Therefore, the results of the present study, which showed an increase in VT in the affected area of the SCP in BRVO eyes, might be more prominent and specific than those of previous studies regarding venous tortuosity in both BRVO and CRVO^8^.

Next, we investigated the association between VA and microvascular parameters. Better VA on the examination day and after 1 year in BRVO was associated with higher VD of the affected area of the DCP, and factors including branch number, sum BL, and sum EL were also associated (Tables 3 and 4). In CRVO, the same factors observed for the DCP as for the affected area of the DCP in BRVO were associated with better VA on the examination day and after 1 year. From these results, good visual outcomes are thought to be associated with a higher number of vessels in the DCP, as previously reported^10^. Interestingly, higher mean BL and EL in the affected area of the DCP were additionally associated with VA 1 year after initial treatment (Table 4). The higher mean BL might reflect the preservation of microvessel structures, and therefore, the eye might have a higher capacity to provide a perfused state to the damaged area after treatment, leading to a good visual outcome after treatment. Another interesting finding is the association between VT and the amount of VA improvement in BRVO eyes. A larger improvement in VA from baseline to 1 year in BRVO eyes was associated with lower VT in the affected area of the DCP (Table 5), although VT did not show a significant association with VA on the examination day or after 1 year. As discussed earlier, lower VT in the DCP is thought to be induced by the decreased transmural pressure derived from worse perfusion. Due to the ceiling effect, anti-VEGF treatment might alleviate the hypoperfused state in eyes with lower VT relatively more than in eyes with higher VT. Therefore, VT of the DCP in BRVO may be a new morphologic factor associated with visual improvement that reflects the major pathogenic mechanism of RVO at the microvascular level. In CRVO, the improvement of VA was associated with higher mean BL in the DCP, and these changes were positively correlated with higher branch numbers (r = 0.59, *P* = 0.01, Fig. 3, Supplementary Table S2) but not with VT (r = 0.38, *P* = 0.09, Fig. 3, Supplementary Table S2). Based on this result, VA improvement was associated with a better perfusion state in CRVO than in BRVO. This result might be from the significantly poorer VA and decreased branch numbers at baseline (1.02 and 282.4, respectively, Tables 1 and 2) in CRVO eyes than in BRVO eyes (0.37 and 318.6, respectively, Tables 1 and 2). Under severe ischemic conditions in CRVO, improvement of VA might occur preferably in the eyes possessing better potency of perfusion, i.e., with abundant branches. Whether the main pathogenic mechanisms underlying BRVO and CRVO are the same is still debatable. However, the differential prognostic factors identified in the present study suggest that some differences in features exist between eyes with BRVO and CRVO.

This study has several limitations. We used images of the maximal 3-mm diameter. Although we gathered 6 mm images, the area outside the central 3 mm square showed various artifacts when clinicians inspected the raw data (H. L and H. C), which leads to a risk of falsely representing the real change. Therefore, our results should not be extrapolated to a wider area, and further studies using larger areas and good image quality should be performed to more clearly understand the changes in RVO. Another limitation is that we did not consider possible diurnal changes in perfusion in the SCP and DCP, although no evidence of diurnal changes in microvascular perfusion was reported in the RVO patients. In our study, OCTA images of 18 eyes (58.1%) among control eyes, 16 eyes (47.1%) among BRVO eyes and 12 eyes (57.1%) among CRVO eyes were acquired between 8:30 AM and 11:30 AM, while other images were acquired between 1:00 PM and 4:00 PM. In a previous study in healthy participants, retinal perfusion in both the SCP and DCP did not differ during the day^11^. Therefore, further study might be needed to homogenize the time of image acquisition to exclude possible diurnal variation in RVO. Finally, because anti-VEGF injection may affect VT^6^, OCTA acquisition at the initial visit or after the first injection in the included eyes would be ideal. However, at the initial visit or the early stage of treatment, improper layer segmentation of the SCP and DCP or artifacts frequently occurred due to ME. Therefore, images with sufficient quality for analysis as close as possible to the initial visit were selected. Future study with an advanced OCT machine that can acquire perfusion status even in eyes with severe ME might overcome this limitation.

In conclusion, various microvascular changes on OCTA of RVO were discovered as new imaging biomarkers. For the improvement of VA over 1 year, lower VT in the affected area of the DCP was associated with VA improvement in BRVO eyes. In CRVO eyes, improvement of VA was associated with higher mean BL and mean EL of the DCP.

## Methods

### Participants

This study followed the Declaration of Helsinki and was approved by the institutional review board of the Konkuk University Medical Center. An exemption was granted from the requirement for informed consent because the study had a retrospective design. We retrospectively reviewed the medical records of 86 subjects, including 55 RVO patients (34 BRVO eyes and 21 CRVO eyes) and 31 healthy control subjects who visited the Department of Ophthalmology, Konkuk University Medical Center, between August 2017 and October 2019. RVO was diagnosed with conventional multimodal imaging: color fundus photography (Optos 200Tx; Optos PLC, Dunfermline, Scotland, UK), FA (Spectralis HRA + OCT; Heidelberg Engineering, Heidelberg, Germany), and SD-OCT (Spectralis HRA + OCT). All the patients underwent a comprehensive ophthalmic examination including measurement of best-corrected VA using the Snellen chart and intraocular pressure, color fundus photography, SD-OCT, and OCTA. Patients received treatment for ME via injections of intravitreal anti-VEGF in the affected eye based on a pro re nata (PRN) regimen.

The exclusion criteria were high myopia (> 6 diopters), images obscured by media opacity, significant cataract progression affecting image quality or causing visual loss, additional concomitant retinal diseases, glaucoma, a history of uveitis, and ocular trauma. We also excluded poor OCTA images with projection artifacts from vessels located above the plane of the image or eye movement artifacts that emerged as a result of disruption of the vessel morphology.

### Imaging OCTA and processing

Spectral-domain OCTA data were acquired with a Spectralis OCT (Spectralis HRA + OCT2; Heidelberg Engineering, Heidelberg, Germany) with a central wavelength of 870 nm and a scan speed of 85,000 A scans per second. The scanned area was 6 × 6 mm, and the distance between the two single B scans in the volume was set to 11 μm. After acquiring the binary image of the 6 × 6-mm area, a 3 × 3-mm square image was cropped to analyze parameters within the macular area. The SCP and DCP were segmented using the inbuilt software of the device. Projection artifacts from DCP vessels were eliminated by a built-in function of the review software (Heidelberg Eye Explorer; Heidelberg Engineering, Heidelberg, Germany). Poor image quality due to ME at the initial visit or the early stage of treatment frequently accompanied segmentation errors of the SCP and DCP layers and image artifacts. Therefore, OCTA images with sufficient quality for analysis as close as possible to the initial visit were selected. OCTA images with good quality without any artifact hindering the image analysis were selected as close as possible to the initial visit. For BRVO eyes, OCTA images with good quality were acquired at the initial visit in 8 eyes (24%) and after 1–4 injections (mean 1.8 injections) in 26 (76%) eyes. For CRVO eyes, OCTA images with good quality were acquired at the initial visit in 4 eyes (19%) and after 1–4 injections (mean 2.3 injections) in 17 (81%) eyes (Table 1).

Eyes with vein occlusion in BRVO and CRVO were named ‘BRVO eyes’ and ‘CRVO eyes’, respectively. Fellow eyes were named ‘fellow eyes of BRVO eyes’ and ‘fellow eyes of CRVO eyes’, respectively. In healthy control subjects, OCTA images of right eyes were selected. In BRVO and CRVO patients, OCTA images of both ‘BRVO eyes’ and ‘CRVO eyes’ and ‘fellow eyes of BRVO eyes’ and ‘fellow eyes of CRVO eyes’ were collected. Quantitative parameters were calculated from processed OCTA images. First, we converted the original images to an 8-bit grayscale image using Fiji (free downloadable software, https://fiji.sc). Vessel binarization was performed using the ‘Trainable Weka Segmentation’ plugin in Fiji (Supplementary Fig. S1A,B). A representative OCTA image was used to train the software to distinguish vessels from the background by drawing representative lines inside and outside the selected vessels^7^. After generating the proper vessel classifier, the whole image was classified, and the result was displayed overlaying the original image. After confirming the adequacy of vessel segmentation in the whole area of the image, the classifier was applied to all other images. Classifiers were created for the SCP and DCP to optimize the vessel binarization of each layer.

In BRVO patients, the acquired OCTA images were further cropped into the superior half and inferior half and assigned as the ‘affected area’ and ‘nonaffected area’ according to the status of vein occlusion. In healthy controls and CRVO patients, acquired OCTA images were also cropped into the superior half and inferior half and assigned as the ‘superior area’ and ‘inferior area’.

From the binarized image, VD was calculated as a proportion of pixels of vessels compared to the total area. The pixels occupied by blood vessels were quantified using the ‘Measure’ function of Fiji.

$$\mathrm{VD }=\frac{\text{Sum\,of\,pixels\,occupied\,by\,blood\,vessels}}{\text{Total\,pixels\,of\,images}}$$

Using the ‘Skeletonize’ plugin of Fiji, binary images were skeletonized, and the skeletonized images had thin tracks of vessels with a 1-pixel diameter (Supplementary Fig. S1C). Using the ‘Analyze skeleton’ plugin in Fiji, the actual length of each branch and the chord length between two branch nodes (EL) were marked and calculated (Supplementary Fig. S1D). VT was calculated as the ratio between the sum of BLs (sum BL) and the sum of chord lengths between branch nodes (sum of ELs, sum EL) (Supplementary Fig. S1E);

$$\mathrm{VT }=\frac{\text{Sum\,of\,branch\,lengths }}{\text{Sum\,of\,Euclidean\,lengths\,bewteen\,branch\,nodes}}$$

Additionally, the mean BL and mean EL were calculated from the data from the skeleton analysis, including the BLs and ELs of small branches, which are subsets of larger vessels. Finally, the number of all small branches was counted (branch number).

### Statistical analyses

The BCVA was converted to the logarithm of the minimal angle of resolution (logMAR) equivalent before statistical analyses. A chi-square test was performed to compare the ratios of sex, diabetes mellitus and hypertension among the five studied groups, including the control eyes, affected and fellow eyes of BRVO patients, and affected and fellow eyes of CRVO patients. Differences in the mean values of parameters among the five studied groups including age, VA and CMT were compared by analysis of variance (ANOVA). The number of injections between the BRVO eye and CRVO eye group was compared by the Mann–Whitney test. Student's *t-*test was used to compare the parameters of affected or fellow eyes with those of the control group. Paired t-tests were performed on the parameters between the affected eyes and their fellow eyes or between the superior area and inferior area in the same eye. Univariate linear regression analyses were performed to evaluate the contribution of each parameter to the VA. *P* < 0.05 was considered statistically significant. SPSS version 18.0 (SPSS, Chicago, IL, USA) was used for all statistical analyses. Graphs were created using Excel 2016 software (Microsoft Corp., Seattle, WA, USA).

## Supplementary information

Supplementary Information.

## Data Availability

The datasets generated during and/or analyzed during the current study are not publicly available due to our hospital’s policy regarding patient records but are available from the corresponding author upon reasonable request.
