# Supplementary material for: Characterization of microvascular tortuosity in retinal vein occlusion utilizing optical coherence tomography angiography
Source: Sci Rep. 2020 Oct 20;10:17788. doi: 10.1038/s41598-020-74871-7 (PMC7576190; doi:10.1038/s41598-020-74871-7)
Supplement: Supplementary file 1 — Supplementary Information. [file 41598_2020_74871_MOESM1_ESM.pdf]

Characterization of Microvascular Tortuosity in Retinal Vein Occlusion Utilizing Optical Coherence Tomography Angiography

Hyungwoo Lee, MD, PhD[1]; Myung Ae Kim, MD[1]; Hyung Chan Kim, MD, PhD[1]; Hyewon Chung, MD, PhD[1]

Department of Ophthalmology, Konkuk University School of Medicine, Konkuk University Medical Center, Seoul, Republic of Korea[1]

Supplementary Table S1. Values of microvascular parameters in the nonstudied, inferior area contralateral to the superior area of the studied eyes.

	Control (N=31)	CRVO fellow (N=21)	CRVO eyes (N=21)
**SCP**			
**Branch number**	286.710 ± 87.711	277.571 ± 107.480	249.524 ± 80.863
**Sum of branch lengths (mm)**	32.650 ± 7.530	31.838 ± 8.885	30.089 ± 8.157
**Sum of Euclidean lengths (mm)**	28.641 ± 6.527	27.768 ± 7.457	26.059 ± 7.029
**Mean branch length (μm)**	117.318 ± 13.754	122.496 ± 25.415	124.070 ± 17.420
**Mean Euclidean length (μm)**	103.022 ± 12.024	107.272 ± 22.936	107.510 ± 15.063
**Vessel tortuosity**	1.139 ± 0.012	1.144 ± 0.018	1.154 ± 0.017
**Vessel density**	0.228 ± 0.040	0.222 ± 0.047	0.211 ± 0.048
**DCP**			
**Branch number**	468.032 ± 96.884	411.857 ± 102.400	278.190 ± 124.784
**Sum of branch lengths (mm)**	38.386 ± 9.210	33.527 ± 10.059	22.538 ± 10.901
**Sum of Euclidean lengths (mm)**	31.711 ± 7.358	27.990 ± 8.178	18.973 ± 8.981
**Mean branch length (μm)**	81.451 ± 4.743	80.223 ± 7.663	79.603 ± 6.932
**Mean Euclidean length (μm)**	70.303 ± 3.849	67.171 ± 6.056	67.288 ± 5.682
**Vessel tortuosity**	1.208 ± 0.019	1.194 ± 0.019	1.183 ± 0.019
**Vessel density**	0.213 ± 0.042	0.190 ± 0.045	0.132 ± 0.058

All values are presented as the mean ± standard deviation.

Control = right eye of healthy subjects; CRVO eyes = eyes diagnosed with central retinal vein occlusion; CRVO fellow = contralateral eye of the eye diagnosed with CRVO; SCP = superficial capillary plexus; DCP = deep capillary plexus.

Supplementary Table S2. Correlations between mean branch length and other vessel parameters.

	Control	BRVO eyes affected	BRVO eyes nonaffected	CRVO eyes
**SCP**				
**Branch number**	-0.831*	-0.811*	-0.838*	-0.805*
**Sum of branch lengths**	-0.709*	-0.708*	-0.788*	-0.733*
**Sum of Euclidean lengths**	-0.711*	-0.706*	-0.786*	-0.729*
**Vessel tortuosity**	-0.276	-0.518*	-0.623*	-0.571*
**Vessel density**	-0.672*	-0.740	-0.801*	-0.721*
**DCP**				
**Branch number**	0.751*	0.505*	0.796*	0.587*
**Sum of branch lengths**	0.852*	0.600*	0.858*	0.722*
**Sum of Euclidean lengths**	0.852*	0.601*	0.860*	0.723*
**Vessel tortuosity**	0.699*	0.498*	0.800*	0.382
**Vessel density**	0.828*	0.577*	0.857*	0.676*

Values are Pearson's correlation coefficient (r).

* $P<0.05$

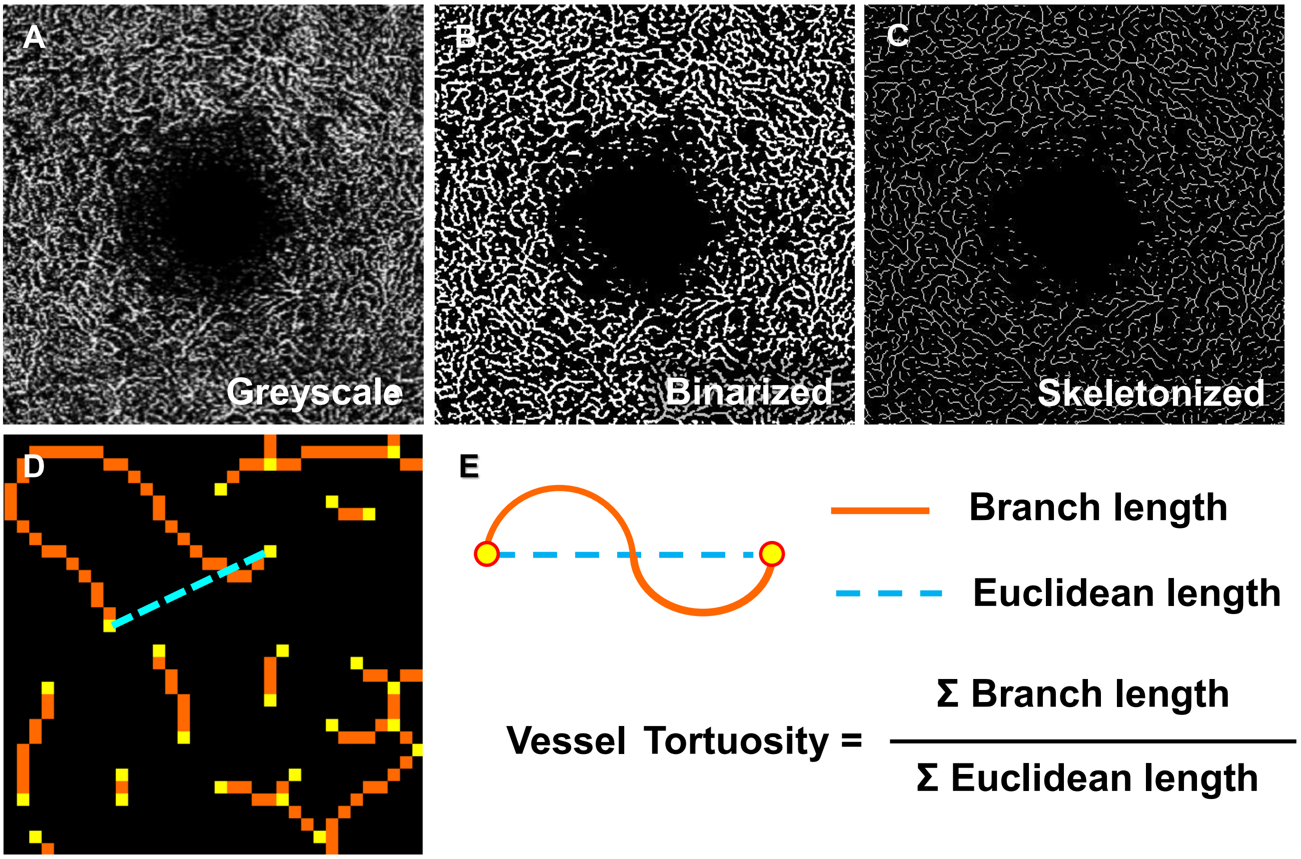

Supplementary Fig. S1. Image processing steps to analyze vessel tortuosity. **(A)** Grayscale OCTA image of the deep capillary plexus in branch retinal vessel occlusion. **(B, C)** The binarized skeletonized image from the original grayscale image. **(D)** The vessel branches (orange) and the branch nodes (yellow) are marked. **(E)** The length of each branch (branch length) and the length of the straight line between two nodes (Euclidean length) were collected by the 'Analyze skeleton' function in FIJI. Based on these data, the mean branch length and mean Euclidean length were calculated. Additionally, vessel tortuosity was calculated as the sum of the branch lengths divided by the sum of the Euclidean length.
